# Comparison of demineralized bone matrix and hydroxyapatite as carriers of *Escherichia coli* recombinant human BMP-2

**DOI:** 10.1186/s40824-021-00225-7

**Published:** 2021-08-03

**Authors:** Yuan-Zhe Jin, Guang-Bin Zheng, Jae Hyup Lee, Shi-Huan Han

**Affiliations:** 1 Department of Orthopedic Surgery, College of Medicine, Seoul National University, Seoul, 03080 South Korea; 2 The First Hospital of Jilin University, Changchun City, 130021 China; 3 Department of Orthopaedics, Taizhou Hospial of Zhejiang Province, Linhai, 317000 Zhejiang China; 4 Department of Orthopedic Surgery, SMG-SNU Boramae Medical Center, 39 Boramae Gil, Dongjak-Gu, Seoul, 156-707 South Korea; 5 Department of Orthopaedic Surgery, Seoul National University, College of Medicine, Institute of Medical and Biological Engineering, Seoul National University Medical Research Centre, SMG-SNU Boramae Medical Centre, Boramae-ro 5-gil 20, Dongjak-gu, Seoul, 07061 South Korea; 6 Department of Orthopedic Surgery, YanBian University Hospital, Yanji, 133000 Jilin Province China

**Keywords:** Bone morphogenetic protein-2, Demineralized bone matrix, Hydroxyapatite, Carrier, Bone regeneration

## Abstract

**Background:**

Autograft has been widely used in various orthopedic and dental surgery for its superior osteogenicity, osteoinductivity and osteoconductivity. But the available volume of the autograft is limited and the efficacy of it is highly affected by the condition of the patients. Therefore, growth factors such as *Escherichia coli* bone morphogenetic protein-2 (ErhBMP-2) has been widely used in some countries and regions with various carriers that could affect the effects of the growth factors. Demineralized bone matrix (DBM) has been widely used as a bone graft substitute and growth factor carrier, but its effect as a carrier of ErhBMP-2 was less investigated.

**Materials and methods:**

Rat calvaria defect model was used in this study. We implanted ErhBMP-2 with DBM or hydroxyapatite (HA) as a carrier in 8 mm calvaria defect and compared their bone regeneration effect in 4th week and 8th week after implantation with micro-CT and histology. The data was analyzed with one-way ANOVA method with Bonferroni post-hoc analysis.

**Result:**

The group with DBM as the carrier showed significantly higher bone volume and bone thickness than the groups with HA as the carrier in both weeks. And the histology sections showed less adipose tissue formed in the groups with DBM as the carrier.

**Conclusion:**

DBM could be a better carrier for ErhBMP-2 than HA.

## Background

Autologous bone graft has been widely used in trauma, spine, and dental surgery to promote bone regeneration [1]. But in the cases of large bone defects, multiple bone harvesting history or metabolic bone diseases, the autologous bone graft could be inadequate for use [2]. Therefore, bone substitutes with growth factors, such as demineralized bone matrix (DBM) with *Escherichia coli* recombinant human bone morphogenetic protein-2 (ErhBMP-2) have been widely used for promoting bone regeneration [3–7]. The ErhBMP-2 has been proved to have comparable osteoinductivity with the mammalian cell derived BMP-2 at both pre-clinical and clinical level, which also had advantage of easy accessing and lower cost [8, 9]. The carriers immobilize the growth factor at the particular site for a sufficient period for inducing bone formation and affects the therapeutic effect of BMP-2 [7]. Hydroxyapatite (HA) is an osteoconductive calcium phosphate ceramic that has a similar chemical structure with inorganic component of bone and have been proved to be a carrier for ErhBMP-2 [8, 10–13]. However, it had low fracture toughness and degradability, and the remnant HA impedes bone remodeling that made it a less optimal carrier [1, 14]. Demineralized bone matrix (DBM) is another BMP-2 carrier, which is both osteoinductive and degradable [15, 16]. It has been practiced over several years for its safety and excellent biocompatibility as both scaffold for bone regeneration and carrier for growth factors [17–19]. But as far as we know, no direct comparison was conducted between the effect of HA and DBM as the carrier of ErhBMP-2. Therefore, we loaded ErhBMP-2 on HA and DBM and compared their effect on bone regeneration with rat calvaria defect model.

## Method

### Rat calvarial defect model

The procedures that involved the use of animals for the rat calvarial defect experiment were approved by the international animal care and use committee (SNUH IACUC No.13–0348). For animal welfare and reduction of animal number, a group of data from a previously published article with the same setting as in this study was used [20]. Eight-week-old male Sprague-Dawley rats (200–220 g, total *N* = 130) were used for the animal experiment, all animals were kept in a 12:12 dark/light cycle, specific-pathogen-free cage and were provided with abundant food and water. The experiments were performed after 1 week of stabilization period. The HA granules with 0.6 mm to 1 mm size were soaked into the 0.15 ml saline containing different dosage of BMP for 5 min. In the groups with DBM as carrier, the BMP was injected into the syringe containing DBM. After the carriers were soaked with BMP-2, the carriers were gently deposited inside the defect. The animals were randomly assigned to following 5 groups: HA (Novosis, CGBio, Korea) 25 mg; DBM 0.05 ml; ErhBMP-2 25 μg (Novosis, CGBio, Korea) + HA 25 mg (Novosis, CGBio, Korea); ErhBMP-2 2.5 μg (Novosis, CGBio, Korea) + HA 25 mg (Novosis, CGBio, Korea); ErhBMP-2 2.5 μg (Novosis, CGBio, Korea) + DBM 0.05 ml (Rafugen DBM gel, Cellmud, Korea). The animals were euthanized in their 4th and 8th weeks after surgery, and each group had 13 animals at each time point.

Animals were anesthetized with intraperitoneal injections of 20 mg/kg Zoletil and 10 mg/kg xylazine. After an 8 mm calvarial defect was made with a high speed trephine burr. The BMP-2 containing carrier was implanted in the defect and the defect was sutured layer by layer [21].

All animals were sacrificed with a CO_2_ chamber under deep anesthesia at their 4th or 8th week after experiments. The calvaria were harvested and fixed in 10% formalin for micro-CT evaluation and histological assessments.

### Micro-CT

The samples were scanned with a Skyscan 1172 micro-CT scanner (Bruker, Belgium) with the following format: pixel size of 11.93 μm, Al filter of 0.5 mm, energy of 70 kV, current of 141 μA, rotation step of 0.4°. The raw images were reconstructed using the NRecon package (Bruker, Belgium) and analyzed with the CT Analyzer software (CT-An, Bruker, Belgium). The threshold values in grayscale of the newly-formed bone were referred to the values of native bone and was set as 110 to 240. Bone morphometric parameters of newly formed bone inside the defect, including percent bone volume (BV/TV), bone surface/volume ratio (BS/BV), trabecular bone pattern factor (Tb.Pf), structure model index (SMI), trabecular bone thickness (Tb.Th), trabecular number (Tb.N), trabecular separation (Tb.Sp), and degree of anisotropy (DA) were analyzed. The data used in the DBM/ErhBMP-2(2.5 μg) group was previously used in another published article [9].

### Histology

The samples were fixed in 10% formalin and sequentially dehydrated in 80 to 100% ethyl alcohol, infiltrated, and embedded in Technovit 7200 resin (EXAKT, Germany). The resin was solidified with a polymerization system (EXAKT, Germany), the hardened resin blocks were sectioned by using a cutting system (EXAKT, Germany) to 200 μm thick slices, and the slices were ground to a thickness of 50 μm by using a grinding system (EXAKT, Germany). The ground slices were stained with hematoxylin and eosin and the stained bone formations in the scaffolds were observed with an optical microscope.

### Statistic

In the micro-CT analysis, the two rhBMP-2 groups were compared with one-way ANOVA method and followed by Bonferroni post-hoc analysis with SPSS 20 (IBM Corp, Armonk, NY: IBM Corp). *P* value less than 0.05 was deemed as statistically significant. The data were presented as mean and standard deviation.

## Result

### Micro-CT

#### Effect of HA and DBM as bone substitute

**Fig. 1:**
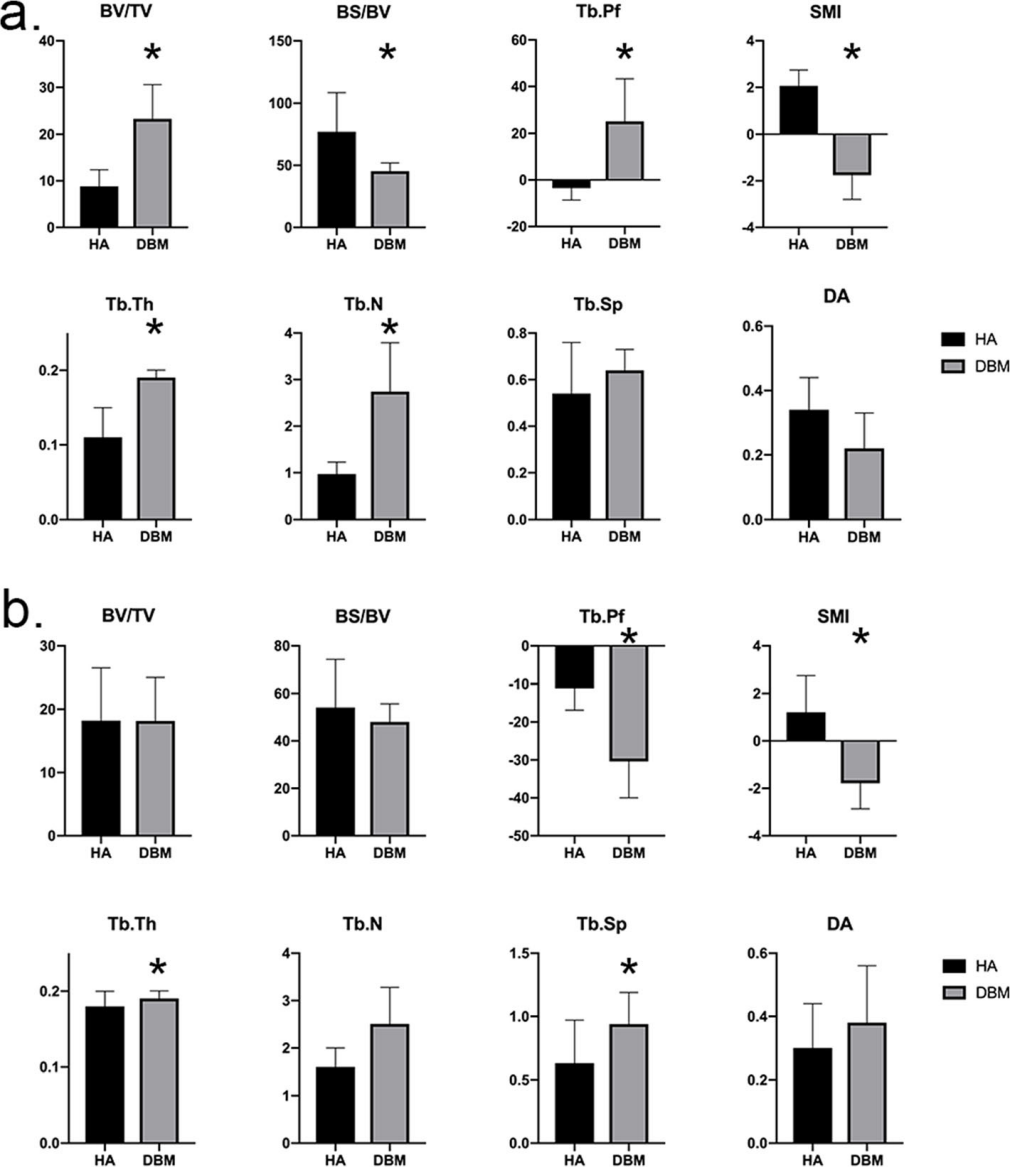
micro-CT results of the HA and DBM groups. **a** Micro-CT results in the 4th week. DBM group had significantly higher BV/TV, Tb. Th, and Tb. N, and significantly lower BS/BV, Tb,Pf and SMI than the HA group. **b** Micro-CT results in the 8th week. The DBM group had similar BV/TV with HA, but significantly higher Tb. Sp and Tb. Th, and significantly lower Tb. Pf and SMI. *, *p* value is less than 0.05

In the 4th week, DBM group had significantly higher BV/TV, Tb. Th, and Tb. N, and significantly lower BS/BV, Tb,Pf and SMI than the HA group. The parameters indicated the new bone in the DBM group had more volume, number, thicker structure, more continuity, more sphere void, and less complex surface than the new bone in the HA group (Fig. 1a).

In the 8th week, the DBM group had similar BV/TV with HA, but significantly higher Tb. Sp and Tb. Th, and significantly lower Tb. Pf and SMI. The parameters indicated the new bone in the DBM group still had more continuity and sphere void, but the percent bone volume became similar to that of HA group. Moreover, the separation between the newly formed bone in the DBM group became significantly wider than that in HA group (Fig. 1b).

#### Effect of HA and DBM as carriers of BMP-2

**Fig. 2:**
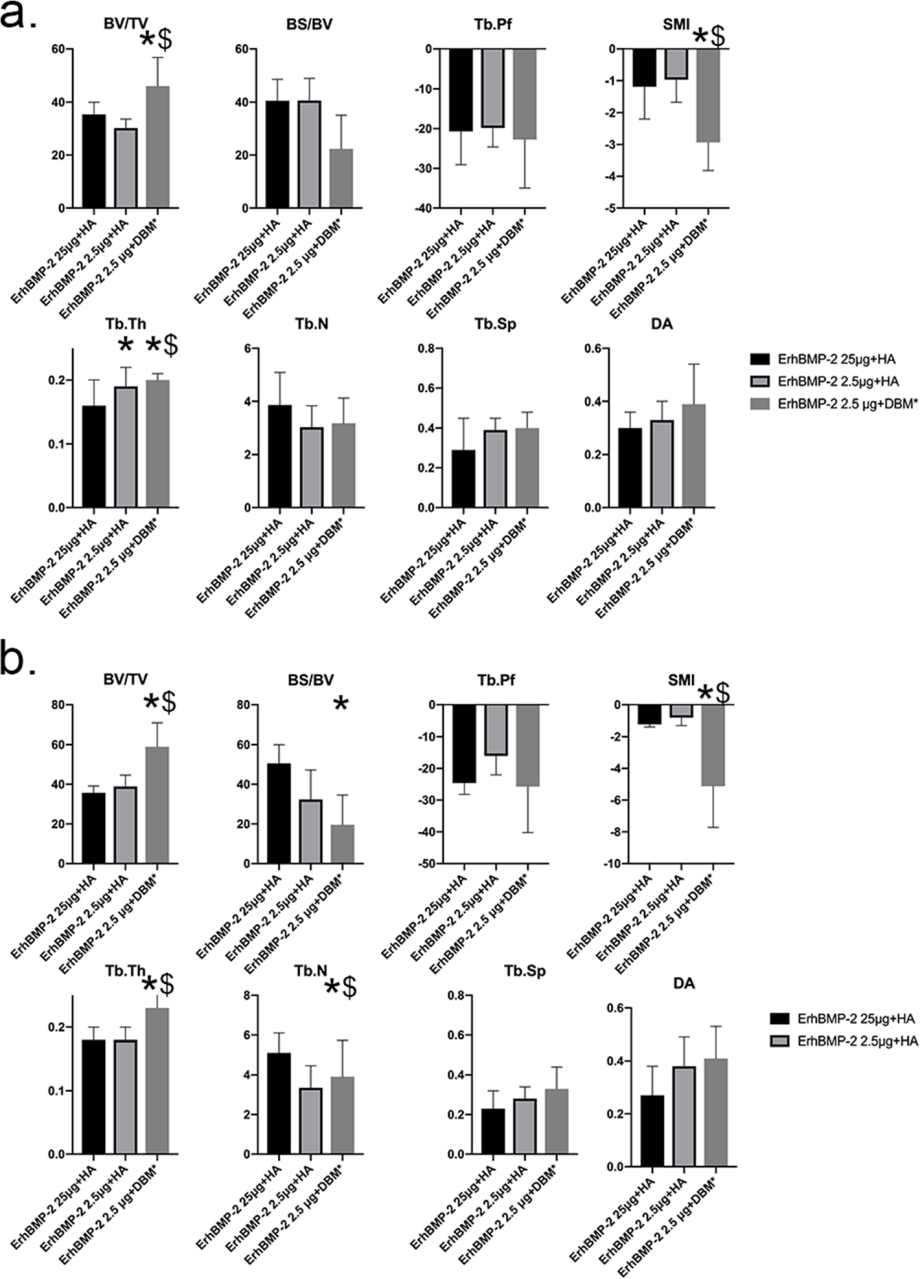
micro-CT results of groups with BMP-2. **a** Micro-CT results in the 4th week. DBM/ErhBMP-2(2.5 μg) group had significantly higher BV/TV and Tb. Th than HA/ErhBMP-2(2.5 μg) group and HA/ErhBMP-2(25 μg) group. Also, the DBM/ErhBMP-2(2.5 μg) group had significantly lower SMI than the other two groups. **b** Micro-CT results in the 8th week. The DBM/ErhBMP-2(2.5 μg) group had significantly higher BV/TV and Tb. Th and significantly lower SMI than the other two groups. *, *p* value is less than 0.05, compare with HA/ErhBMP-2(25 μg) group. ^$^, *p* value is less than 0.05, compare with HA/ErhBMP-2(2.5 μg) group

In the 4th week, DBM/ErhBMP-2(2.5 μg) group had 52 and 30% higher BV/TV than HA/ErhBMP-2(2.5 μg) group and HA/ErhBMP-2(25 μg) group, with statistical significance (Fig. 2a) Also, DBM/ErhBMP-2(2.5 μg) group had significantly higher Tb. Th than the HA/ErhBMP-2(2.5 μg) group and HA/ErhBMP-2(25 μg) group without significant difference in Tb. N or Tb.Sp. The significantly lower SMI in DBM/ErhBMP-2(2.5 μg) group indicated a more spherical structured bone formed in the DBM/ErhBMP-2(2.5 μg) group.

In the 8th week, the BV/TV of DBM/ErhBMP-2(2.5 μg) group was 52 and 65% higher than that of HA/ErhBMP-2(2.5 μg) group and HA/ErhBMP-2(25 μg) group, with statistical significance. Consistent with the result in the 4th week, the DBM/ErhBMP-2(2.5 μg) group had significantly higher Tb. Th than the HA/ErhBMP-2(2.5 μg) group and HA/ErhBMP-2(25 μg) group. The significantly lower SMI in DBM/ErhBMP-2(2.5 μg) group indicated more spherical structured bone formed in the DBM/ErhBMP-2(2.5 μg) (Fig. 2b).

**Fig. 3:**
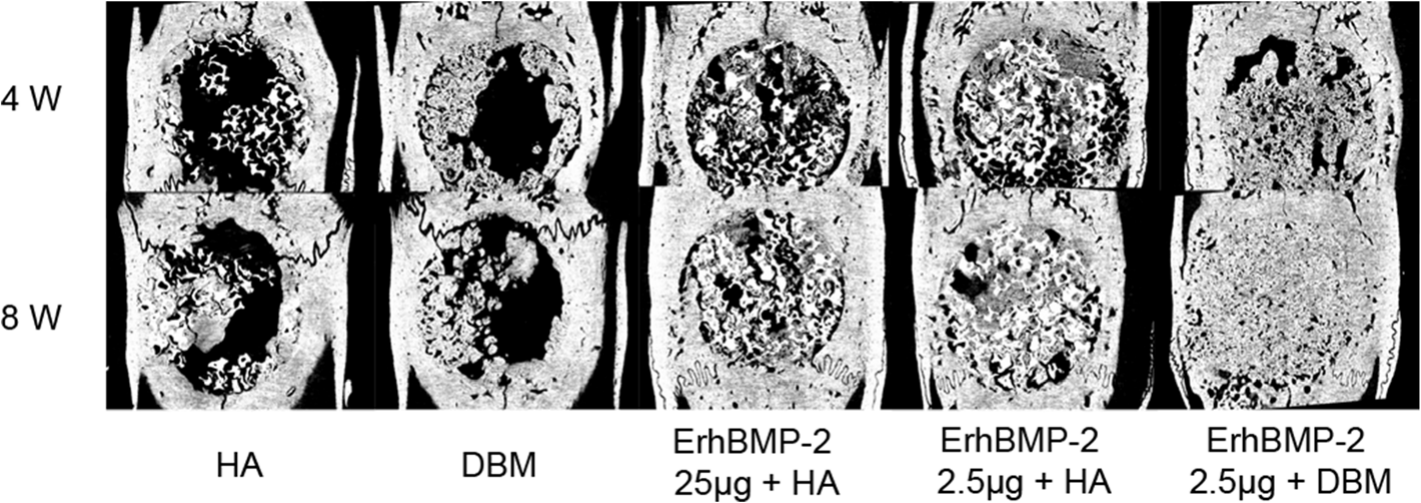
micro-CT images. ErhBMP-2 2.5 μg + DBM had the best bone regeneration in 4th and 8th week. No significant difference was observed between ErhBMP-2 2.5 μg + HA and ErhBMP-2 25 μg + HA at both time points. DBM group achieved faster bone regeneration than HA in the 4th week, but had similar bone formed with HA group in the 8th week. The data in DBM + ErhBMP-2 group was reported in a previous article [20]

The parameters indicated the bone formed DBM/ErhBMP-2(2.5 μg) group maintained its advantage in bone volume, and the bone had more sphere void and more continuity structure. Additionally, the Tb. Th was slightly but significantly higher in DBMErhBMP-2(2.5 μg) group in the 4th week, while in the 8th week, the Tb. Th of DBM/ErhBMP-2(2.5 μg) group became 28% higher than that of the other two groups. The results consistently showed the DBM/ErhBMP-2(2.5 μg) group achieved the best bone regeneration at both time points (Fig. 3).

#### Histology

The histology sections showed consistent with micro-CT result. In the 4th week, the DBM group showed more bone than HA. The HA/ErhBMP-2(25 μg) group had slightly more bone than HA/ErhBMP-2(2.5 μg) group. The DBM/ErhBMP-2(2.5 μg) group had the most bone formed. Additionally, the bone in DBM/ErhBMP-2(2.5 μg) group showed denser construct and less fatty tissue formed inside the newly generated bone than the other groups (Fig. 3).

**Fig. 4:**
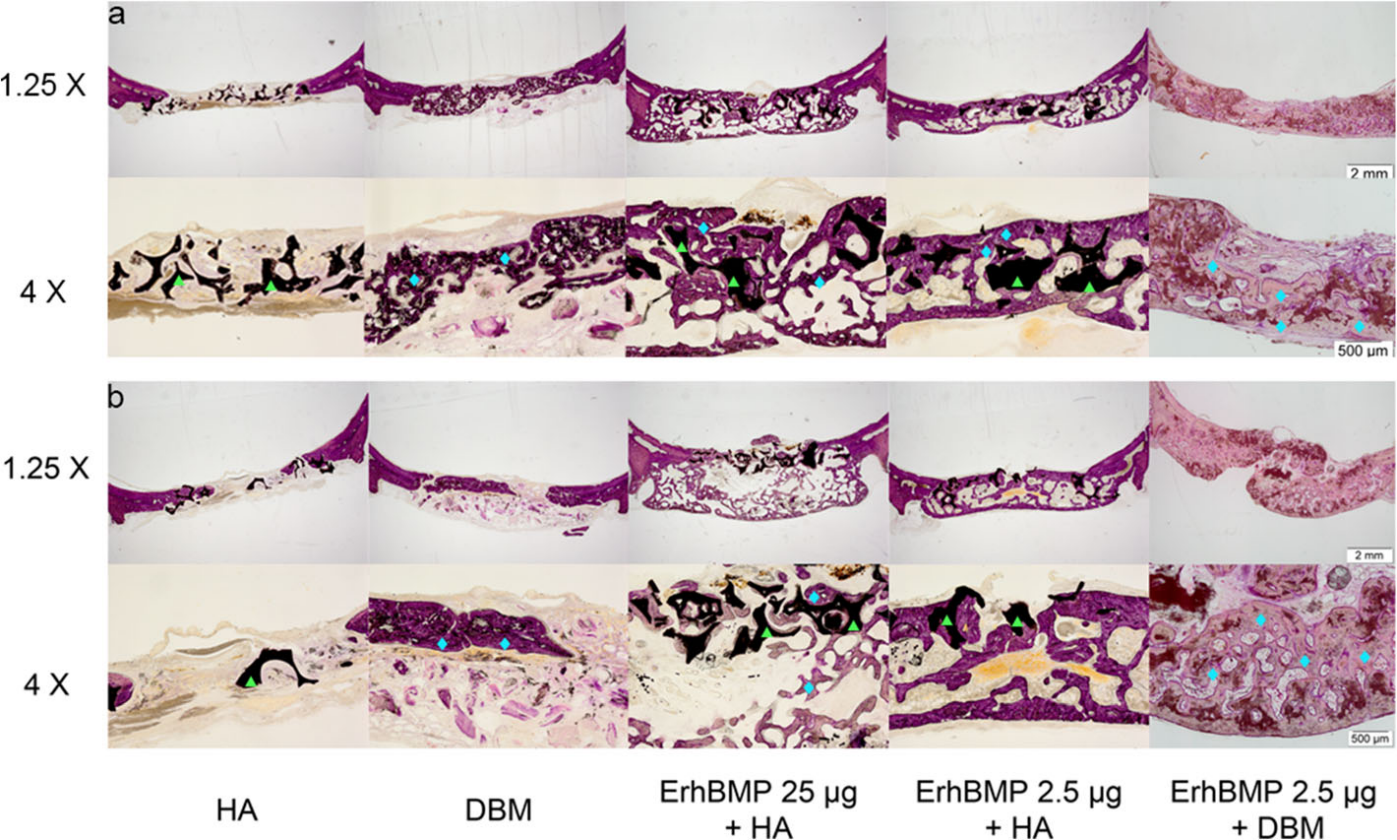
Histology sections. **a** Histology sections in the 4th week. ErhBMP-2 2.5 μg + DBM showed significantly more solid bone regeneration, less adipose tissue formation than ErhBMP-2 25 μg + HA and ErhBMP-2 2.5 μg + HA groups. In both groups with HA as carrier, cyst-like bone and abundant adipose tissue formed in the defect. **b** Histology sections in the 8th week. ErhBMP-2 2.5 μg + DBM group still had the most bone regeneration. ErhBMP-2 25 μg + HA had more adipose tissue formed inside the defect and more hollow structure compared with 4th week. The bone in ErhBMP-2 2.5 μg + HA had less cyst-like bone and adipose tissue than ErhBMP-2 25 μg + HA group had. The DBM group had less bone compared with its 4th week. The green triangles indicated the HAP granules and the cyan diamonds indicated the newly formed bone. The data in DBM + ErhBMP-2 group was reported in a previous article [20]

In the 8th week, HA and DBM showed similar bone regeneration. HA/ErhBMp-2(25 μg) group showed similar bone volume and more sphere-shaped void with HA/ErhBMP-2(2.5 μg) group. The DBM/ErhBMP-2(2.5 μg) group had the most bone generated in the defect, and the bone was the thickest among the groups. Also, the DBM/ErhBMP-2(2.5 μg) group had the least fatty tissue in the newly formed bone (Fig. 4).

## Discussion

Critical bone defect that cannot heal spontaneously requires bone grafting to provide sufficient endogenous regeneration. The standard grafting material is still the autologous bone, despite its complications. Following the development in the tissue engineering, the ErhBMP-2 has been used in the clinic for promoting bone regeneration [8, 22]. Despite the inspired outcomes in pre-clinical studies, the effectiveness of BMP-2 in clinical cases still need to be furtherly proved, such as non-union. One possible reason is the fast release from the used carrier that mostly determines the effect of the BMP-2, therefore, choosing an appropriate carrier for the ErhBMP-2 remains to be an active area of research. DBM is an attractive bone substitute that can release the BMP-2 with a pattern of a 4-day burst release followed with a 14-day continuous release [16, 23].

The effect of DBM as the carrier was compared in vivo with HA, another widely used bone substitute with a rapid BMP-2 releasing pattern [24]. In the comparison of mere DBM and HA, the micro-CT showed similar bone regeneration on the 8th week. But with BMP-2 added, the DBM showed significantly and obviously more bone regeneration at both time points compared with its same dosage counterpart. The possible reasons might be the growth factors that already contained in the DBM that ErhBMP-2 synergistically elevated bone regeneration with ErhBMP-2 and its superior transfer rate and releasing pattern of BMP-2 [19]. More surprisingly, we noticed even adding 10 times dosages of BMP-2 in HA did not significantly increased bone regeneration at both time points, which furtherly proved merely increasing the dosage of ErhBMP-2 cannot guarantee a better bone regeneration.

In previous studies, it has been attempted to optimize the release of rhBMP-2 from carrier to achieve better bone regeneration. Zhu et al. investigated a collagen-binding rhBMP-2, and this structure modified rhBMP-2 achieved significantly slower release and better bone formation than the commercial BMP-2 while both with DBM as a carrier [17]. Also, a heparin conjugated carrier system was proved to reduce adipose tissue formation and enhance bone generation by ErhBMP-2 [25, 26]. However, the new product listed above remained in preclinical experiment phases and needed further investigation of their safety and effect character. Compare with the newly fabricated material and modified rhBMP-2, the HA, DBM, and rhBMP-2 used in this study were all commercially available. Therefore, the result from this study might offer more practical evidence for using ErhBMP-2 in a clinical situation and provide a possible combination of ErhBMP-2 and its carrier system.

On the other side, though DBM/ErhBMP-2(2.5 μg) performed the best bone regeneration, DBM itself might not be an ideal carrier. Firstly, all DBM clinical product is from a human donor. Though donor qualification process is rigorous and stringent, using DBM still has a risk of disease transmission [15]. Moreover, the efficiency could also be affected by donor variability, like age and gender [27]. Secondly, the product of DBM was in powder shape and it requires other carriers for handling, and the carriers used to mix DBM could bring variety in the final efficacy [27].

In selecting the bone defect model, we used a rat calvaria defect model, which has been widely used for generating standardized defects [28]. But it cannot simulate all bone defect diseases in a clinical situation. Because the calvaria was composed of flat bone and healed through intramembranous ossification, and it cannot be used to assess the material under physiological mechanical loads [28, 29]. The micro-CT analysis might overestimate the bone formation in groups with HA due to the difficulties in separating HA from newly formed bone. But even considering the possible overestimated bone volume in HA group, the groups with DBM as the carrier showed higher bone volume. Therefore, with the consistent trend observed from histology sections, we believe the result from micro-CT could be considered reliable. In current stage of study, the release kinetics, transfer rate or the degradation characteristics of the two carriers were not investigated, which brought difficulties in furtherly interpreting the reasons of the superior effect of the DBM to HA. In further studies, the reasons of the better performance of DBM should be elucidated. At current stage of study, the surface morphology of the DBM or HA was not obtained, which brought hassles in analyzing the possible reasons of the result and was a limitation of this study. Additionally, the histomorphometry analysis result should be performed to bring more concise results. The performance of DBM as a carrier of ErhBMP-2 should be evaluated with defect models that can simulate spinal fusion and long bone defect, in which bone regenerates through endochondral ossification, blood flow might dilute the dosage of ErhBMP-2 and mechanical stress could be load [28, 29].

## Conclusion

DBM presented a potent carrier of ErhBMP-2 the induced significantly better bone regeneration than HA. The combination of DBM and ErhBMP-2 that could be a feasible bone substitute for augmentation of bone defect.

## Data Availability

The data that support the findings of this study are available from the corresponding author, JHL, upon reasonable request.

## Abbreviations

BV/TV: Percent bone volume
BS/BV: Bone surface/volume ratio
DA: Degree of anisotropy
DBM: Demineralized bone matrix
ErhBMP-2: Escherichia recombinant human bone morphogenetic protein-2
HA: Hydroxyapatite
SMI: Structure model index
Tb.Pf: Trabecular bone pattern factor
Tb.Th: Trabecular bone thickness
Tb.N: Trabecular number
Tb.Sp: Trabecular separation
